# Best evidence summary for nutritional management of cancer patients with chyle leaks following surgery

**DOI:** 10.3389/fnut.2024.1478190

**Published:** 2025-01-08

**Authors:** Jie Zhou, Wentao Huang, Ya Hu, Fen Liu, Man Xu, Xiaoping Chen, Mingzhu Xin, Huiming Lu, Xia Zheng

**Affiliations:** ^1^Department of Urinary Surgery, State Key Laboratory of Oncology in South China, Guangdong Key Laboratory of Nasopharyngeal Carcinoma Diagnosis and Therapy, Guangdong Provincial Clinical Research Center for Cancer, Sun Yat-sen University Cancer Center, Guangzhou, China; ^2^Postanesthsia Care Unit, Department of Anesthesiology, State Key Laboratory of Oncology in South China, Guangdong Key Laboratory of Nasopharyngeal Carcinoma Diagnosis and Therapy, Guangdong Provincial Clinical Research Center for Cancer, Sun Yat-sen University Cancer Center, Guangzhou, China; ^3^Department of Nursing, State Key Laboratory of Oncology in South China, Guangdong Key Laboratory of Nasopharyngeal Carcinoma Diagnosis and Therapy, Guangdong Provincial Clinical Research Center for Cancer, Sun Yat-sen University Cancer Center, Guangzhou, China

**Keywords:** chyle leaks, lymph node dissection, cancer, nutritional management, best evidence, review

## Abstract

**Background:**

Chyle leaks (CL) is a significant postoperative complication following lymph node dissection in cancer patients. Persistent CK is related to a series of adverse outcomes. Nutritional management is considered an effectively strategy that treat CL. However, the existing evidence on nutritional management for this patient cohort fails to provide actionable clinical guidance.

**Aim:**

This study was aimed to establish an evidence-based framework for nutritional management, offering reliable basis for clinical nursing practice.

**Methods:**

Utilizing the “6S” mode, we conducted a systematic search of UpToDate, BMJ, Best Practice, Cochrane Library, Joanna Briggs Institute (JBI) Center for Evidence-Based Health Care Database, National Guideline Clearinghouse (NGC), Guidelines International Network (GIN), National Institute for Health and Care Excellence (NICE), Scottish Intercollegiate Guidelines Network (SIGN), Registered Nurses' Association of Ontario (RNAO), World Health Organization, Medlive, American Society for Parenteral and Enteral Nutrition (ASPEN), European Society for Clinical Nutrition and Metabolism (ESPEN), Web of Science, PubMed, Embase, CINAHL, China Biology Medicine (CBM), and China National Knowledge Infrastructure (CNKI) for all evidence on the nutritional management of postoperative coeliac leakage in cancer patients. This search included guidelines, evidence summaries, expert consensus, clinical decision-making, recommended practices, systematic evaluations or Meta-analyses, randomized controlled trials (RCTs), and class experiments. The search timeframe was from the library's establishment to June 2024. Quality assessment of the literature was completed independently by two researchers with professional evidence-based training and expert advice, and evidence was extracted and summarized for those that met the quality criteria.

**Results:**

A total of 13 articles were included in the analysis, comprising two expert consensus, one guideline, one class of experimental studies, seven systematic evaluations, and two clinical decisions. We summarized 22 pieces of evidence across five categories: nutritional screening, assessment, and monitoring, timing of nutritional therapy, methods and approaches to nutritional therapy, nutrient requirements, and dietary modification strategies.

**Conclusion:**

This study presents key evidence for nutritional management in cancer patients with CL post-surgery, emphasizing nutritional screening, assessment, timing and methods of therapy, and dietary adjustment strategies. It emphasized the necessity of thorough screening tools for the assessment of nutritional condition, and the benefits of early enteral feeding. A multidisciplinary team approach is vital for conducting personalized dietary, while sustained nutritional support, dietary fat restrictions, and medium-chain triglycerides enhance nutrient absorption. Consistent monitoring of chylous fluid output and timely dietary adjustments are crucial for improving patient outcomes and recovery.

**Systematic review registration:**

http://ebn.nursing.fudan.edu.cn/registerResources, identifier ES20244732.

## 1 Introduction

Global cancer center statistics reveal a significant surge in cancer incidence and mortality rates. In 91 out of 172 countries, cancer is the leading cause of death among individuals under 70 years old (1). China accounts for 23.7% of new cancer cases and 30% of global cancer deaths. As China progresses into a “deep aging society,” cancer stands as the second leading cause of death among the elderly (≥60 years old). The increasing burden on cancer patients highlights the need for immediate and effective cancer prevention and control measures in China (2).

Surgical resection is the primary modality for cancer treatment, providing definitive therapeutic benefits. Given the frequent occurrence of tumors with lymph node metastasis, intraoperative lymph node dissection is integral to surgical oncology (3–5). Excessive trauma during vessel and tissue separation, incomplete ligation of injured lymphatic vessels, or inadequate heat application from the electrosurgical knife can cause intraoperative lymphatic vessel damage. This damage impedes lymphatic fluid reflux, leading to lymphatic leakage, known as chyle leaks (CL), a significant postoperative complication following lymph node dissection in cancer patients (6).

CL is characterized by milky or white drainage fluid with triglyceride levels ≥ 110 mg/dL (1.2–1.4 mmol/L) (7). Chyle comprises lymphatic fluids enriched with protein, fat, and immunoglobulins. Persistent leakage can lead to impaired wound healing, immunosuppression, malnutrition, extended hospitalization, and occasionally death (8). The hypermetabolic state in cancer patients heightens their nutritional demands, and the nutritional depletion from CL exacerbates this condition. Nutritional management for patients experiencing CL is essential, as it not only addresses the immediate nutritional deficiencies caused by the leakage but also plays a significant role in promoting overall recovery, strengthening immune function, and improving quality of life. Implementing effective dietary strategies can help alleviate various complications associated with CL, such as malnutrition and prolonged length of hospitalization, and can lead to better patient outcomes and help reduce healthcare costs, ultimately benefiting both patients and the healthcare system as a whole (9–11). Studies have shown that oral medium-chain fatty acid complete nutrition reduces chylous fluid. Medium-chain fatty acids are absorbed directly into the portal vein through small intestinal cells, minimally affecting gastrointestinal lymph fluid production, thereby improving chyle color and decreasing the drainage fluid volume (9). This evidence indicates that dietary management can meet the nutritional needs of cancer patients and effectively treat CL (10, 11).

Currently, systematic reviews addressing management strategies for cancer patients with CL post-surgery are sparse, both domestically and internationally, mainly focusing on CL risk factors and treatment (7, 12). The fragmented and non-specific nature of existing evidence on nutritional management for this patient cohort fails to provide actionable clinical guidance. The summary of best evidence is a crucial part of evidence-based nursing, as it synthesizes the highest quality and most relevant research findings to provide clear, actionable insights that can inform clinical decision-making (13). Therefore, the present study systematically gathered and summarized evidence on post-surgical nutritional management of CL in cancer patients, utilizing evidence-based methodologies to create a comprehensive summary. The aim was to establish an evidence-based framework for nutritional management, offering reliable basis for clinical nursing practice.

## 2 Materials and methods

### 2.1 Establishment of problems

The evidence-based problem was constructed using the PIPOST model (14), with the following components: P (target population): cancer patients with CL post-surgery; I (intervention): nutritional management strategies; P (professionals applying evidence): healthcare professionals; O (outcome): CL volume, nutritional status, and physical function; S (site of evidence application): oncology ward; T (evidence type): published guidelines in Chinese and English, evidence summaries, expert consensus, clinical decision-making tools, recommended practices, systematic reviews, meta-analyses, randomized controlled trials (RCTs), and quasi-experiments. This study was registered at the Fudan University Center for Evidence-Based Nursing under registration number ES20244732. This study is a secondary analysis of the existing literature and is considered exempt from ethical review.

### 2.2 Search strategy

Utilizing the “6S” evidence pyramid model, the terms “cancer,” “chyle leak,” “nutrition,” and related keywords were used to query pertinent Chinese and English guideline websites, professional society websites, and the UpToDate Clinical Decision Support Website. For English databases, search terms included “cancer/carcino^*^/tumo^*^/neoplasm^*^/onco^*^/chyle/chyle leakage/chylous fistula/chyle fistula/chylous ascites/chyle leaks/chylothorax/milk leakage/nutrition^*^/nutrition Status/Nourishment/nutrition management/nutritional support/diet therapy/diet/enteral nutrition/enteral feeding/tube feeding/parenteral nutrition/nutrition disorders.” In Chinese databases, terms encompassed “cancer/tumor/chyle leaks/chyle fistula/chylous ascites/chyle/nutrition/nutritional management/nutritional intervention/diet therapy/diet.” The search covered UpToDate, BMJ, Best Practice, Cochrane Library, Joanna Briggs Institute (JBI) Center for Evidence-Based Health Care Database, National Guideline Clearinghouse (NGC), Guidelines International Network (GIN), National Institute for Health and Care Excellence (NICE), Scottish Intercollegiate Guidelines Network (SIGN), Registered Nurses' Association of Ontario (RNAO), World Health Organization, Medlive, American Society for Parenteral and Enteral Nutrition (ASPEN), European Society for Clinical Nutrition and Metabolism (ESPEN), Web of Science, PubMed, Embase, CINAHL, China Biology Medicine (CBM), and China National Knowledge Infrastructure (CNKI). The search period spanned from each database's inception to June 2024, with the PubMed search strategy detailed in Supplementary material.

### 2.3 Study inclusion and exclusion criteria

Inclusion criteria: (1) cancer patients aged ≥ 18 years with CL post-surgery; (2) research on nutritional management for postoperative CL; (3) evidence types: guidelines, evidence summaries, expert consensus, clinical decision-making, recommended practices, systematic reviews, meta-analyses, RCTs, and quasi-experiments; (4) outcome measures: nutrition-related metrics, drainage volume, and length of hospital stay; (5) evidence application site: oncology wards; (6) publication language: Chinese or English. Exclusion criteria: (1) incomplete literature information or duplicates; (2) inaccessible full texts; (3) studies failing quality assessment; (4) outdated guidelines or consensus documents with available updated versions; (5) lower-level evidence incorporated in higher-level evidence.

### 2.4 Literature quality assessment

The study reviewed diverse literature types, including clinical decision-making frameworks, guidelines, systematic reviews, expert consensus, and quasi-RCTs, employing tailored quality assessment tools for each category. (1) Clinical decision-making evidence, lacking an internationally recognized assessment tool, was directly classified as high-quality evidence when sourced from authoritative databases, as indicated in the relevant literature (15). (2) The guidelines were evaluated using the Appraisal of Guidelines for Research and Evaluation (AGREE II) (16), updated in 2012 in the United Kingdom. This instrument includes six dimensions and 23 items, each rated on a scale from 1 to 7, ranging from “strongly disagree” to “strongly agree.” Higher scores indicate better compliance with the item. (3) Systematic reviews, expert consensus, and quasi-RCTs were evaluated based on the criteria of the authentic assessment tool (2016) (17) from the Australian JBI Center for Evidence-Based Health Care. Two researchers, trained in evidence-based methodologies (completing 45 class hours of hospital-conducted evidence-based course training and passing project defense assessments), independently assessed the literature quality according to the evaluation criteria. In cases of disagreement, a third researcher mediated to reach a consensus.

### 2.5 Evidence summary and grading

Two researchers systematically reviewed the literature to extract and summarize evidence. Complementary and consistent content was summarized based on logical coherence, while conflicting content was evaluated with priority given to high-quality, evidence-based, and recent authoritative sources. For example, evidence on nutritional therapy methods cited an expert consensus recommending collaboration with nutritionists to develop individualized dietary regimens and closely monitor clinical responses (18). Another recommendation advocated for a multidisciplinary team (MDT) approach, integrating insights from surgeons, nurse specialists, and nutritionists (19). These aligned recommendations were consolidated. The 2014 JBI evidence pre-grading and recommendation level system was applied to classify the evidence into five levels (20).

## 3 Results

**Figure 1:**
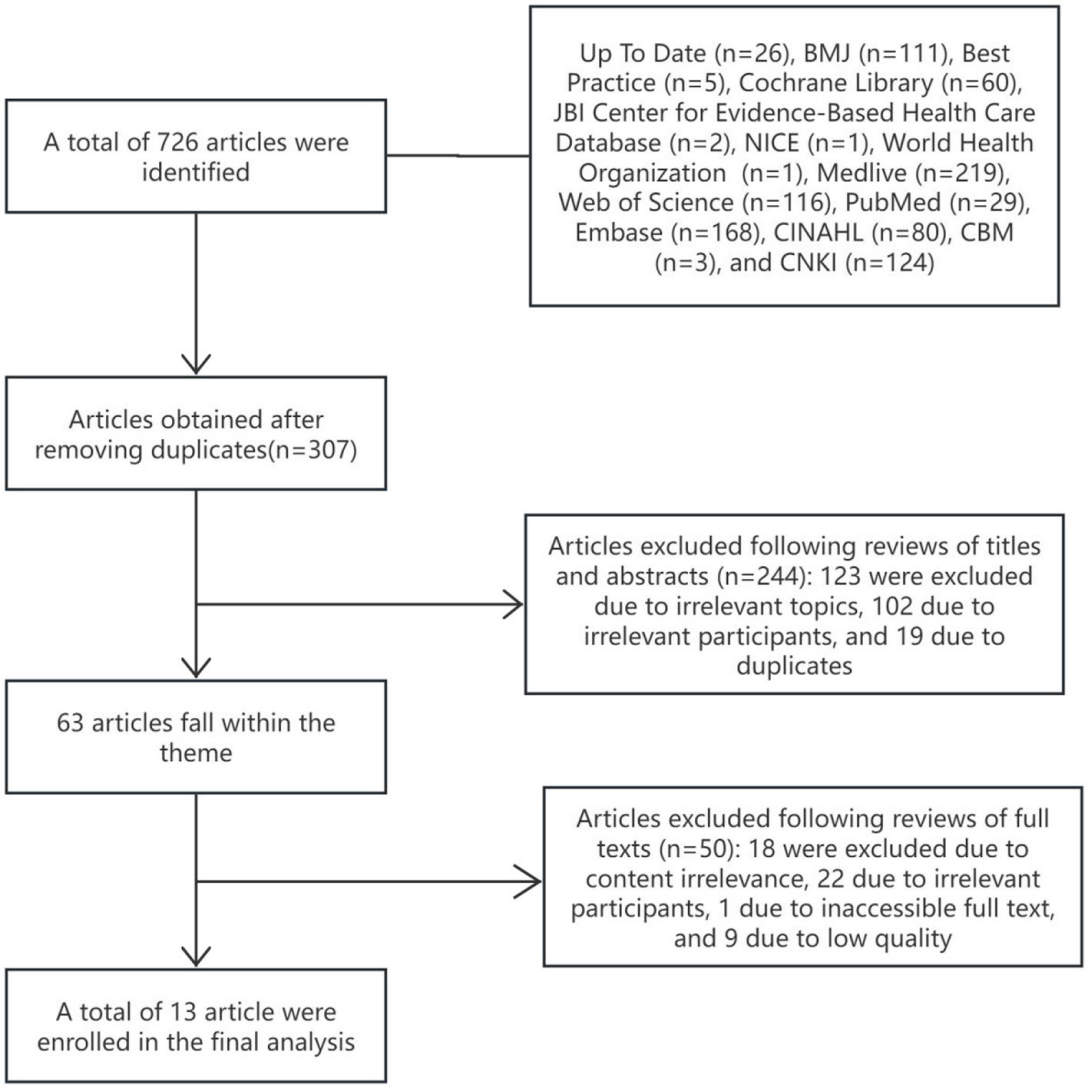
Literature screening flowchart.

The initial search identified 726 studies, with 13 meeting the criteria for inclusion (18, 19, 21–31). The screening process is outlined in Figure 1, and Table 1 provides a summary of the basic characteristics of the selected studies.

**Table 1:** Basic characteristics of the included articles (*n* = 13).

**References**	**Published year**	**Source**	**Subject**	**Type**
He et al. (18)	2022	Medlive	Chinese expert consensus on clinical practice of prevention and treatment of chyle leak in neck dissection of thyroid cancer (2022 Edition)	Expert consensus
Farkas et al. (19)	2020	Pubmed	Management of CL after lymph node dissection	Systematic review
Muscaritoli et al. (21)	2021	ESPEN	ESPEN Practice Guidelines: Clinical Nutrition in Cancer	Guidelines
Zeng et al. (22)	2021	CNKI	Feasibility study of medium-chain fatty acids in head and neck tumor patients with CL following cervical lymph node dissection	Quasi-RCT
Steven and Carey (23)	2015	CINAHL	Nutritional management of CL patients: A systematic review	Systematic review
Muzzolini et al. (24)	2021	Pubmed	Occurrence and risk factors of CL after pancreatic cancer surgery: a systematic review	Systematic review
Varghese et al. (25)	2022	Pubmed	Incidence and risk factors of CL post-pancreatic cancer surgery	Systematic review
Ng et al. (26)	2021	Pubmed	Incidence, clinical manifestations, risk factors, and management strategies of CL after colorectal cancer surgery	Systematic review
Kamarajah et al. (27)	2022	Pubmed	Risk factors, diagnosis, and treatment of CL post-esophageal cancer surgery	Expert consensus
Molena et al. (28)	2021	Embase	Comparison of octreotide and oral dietary adjustment in treating CL post-neck dissection: a systematic review and meta-analysis	Systematic review
Smith et al. (29)	2024	Embase	Nutritional management of CL after head and neck cancer surgery	Systematic review
Heffner (30)	2024	UpToDate	Treatment of chylothorax	Clinical decision-making
Cárdenas et al. (31)	2024	UpToDate	Etiology, classification, and treatment of CL and chylous ascites	Clinical decision-making

This study incorporated two expert consensus articles (18, 27). He et al. (18) received an “unclear” rating for item 6, “Are the views proposed inconsistent with previous literature?” Conversely, Kamarajah et al. (27) achieved a “yes” for all evaluated items, signifying high quality and justifying their inclusion.

One guideline (21) reported AGREEII standardization percentages as follows: 91.23% for scope and purpose, 92.38% for participants, 87.45% for rigor of formulation, 100% for clarity and readability, 82.11% for applicability, and 95.74% for editorial independence. Six domains met the ≥60% threshold, while another six met the ≥30% threshold. The intraclass correlation coefficient (ICC) for evaluation consistency among the three researchers was 0.71, indicating high reliability and justifying inclusion.

In one quasi-RCT (22), all items, except item 6, “Is the follow-up complete? If not, are measures taken?”, received a “yes” rating, whereas item 6 was rated as “unclear.” The study's overall quality was deemed high, warranting its inclusion.

Seven systematic reviews were included (19, 23–26, 28, 29). Items 5, “Are the literature quality assessment criteria used appropriate?” and 6, “Is the literature quality assessment independently completed by two or more reviewers?” were rated as “unclear.” In the studies by Farkas et al. (19) and Ng et al. (26), all evaluation items received a “yes” rating, except for items 5 and 6, which were rated as “unclear.” Similarly, in the studies by Steven and Carey (23) and Molena et al. (28), all evaluation items received a “yes” rating, except for item 9, “Has the possible publication bias been assessed?”, which was rated as “unclear.” All evaluation items in the studies by Muscaritoli et al. (21) and Varghese et al. (25) were rated “yes,” with item 9 rated “unclear.” In the study by Smith et al. (29), all items were rated “yes” except for item 5, “Are the literature quality assessment criteria used appropriate?”, item 6, “Is the literature quality assessment completed independently by two or more evaluators?”, and item 9, “Is the possible publication bias assessed?”, which were rated “unclear.” Due to the high quality and comprehensive design of these studies, they were included. Two clinical decision-making articles from UpToDate (30, 31) were also directly incorporated.

A comprehensive literature review, focusing on clinical relevance and feasibility, identified 22 high-quality evidence pieces across five domains, subsequently graded and summarized in Table 2.

**Table 2:** Best evidence summary for nutritional management of CL post-cancer surgery.

**Evidence topic**	**Evidence description**	**Level of evidence**
1. Nutritional screening, assessment, and monitoring	1. Patients with a low Prognostic Nutritional Index (PNI) exhibit a higher incidence of postoperative CL (25).	3c
	2. The Nutrition Risk Screening 2002 (NRS 2002) and the Patient-Generated Subjective Global Assessment (PG-SGA) are recommended diagnostic tools for assessing nutritional status in cancer patients (21).	5b
	3. Nutritional assessment methods for CL patients include monitoring changes in weight, triceps skinfold thickness, middle-arm circumference, and grip strength (23).	4c
	4. During nutritional support for patients with persistent CL, monitoring hepatorenal function, immune status, and electrolyte levels is recommended (18, 22, 26, 29).	4a
	5. Early enteral feeding has been identified as an independent risk factor for the onset of CL (24, 25).	4a
	6. Evaluating cost, taste adaptation, patient compliance, and potential uncertainties in treatment outcomes is recommended for dietary management of CL patients (19).	4a
2. Timing of nutritional therapy	1. Dietary therapy should be preferred upon the diagnosis of CL (18, 19, 24, 27, 28, 30, 31).	4a
	2. Nutritional therapy is recommended to be maintained throughout the entire CL treatment process (23, 24, 30).	2c
	3. Following resolution of CL through dietary therapy, it is recommended to adjust the diet for 2–4 weeks to prevent recurrence (26).	4a
3. Methods and approaches of nutritional therapy	1. Collaboration with nutritionists, physicians, and other MDTs is recommended to develop a personalized dietary management regimen. Patients should be instructed to follow a high-protein, low-fat diet (< 10 grams of fat/day), with close monitoring of clinical responses and ongoing assessment of nutritional status (18, 19, 26, 29, 30).	2b
	2. Dietary therapy can be performed either alone or in combination with other non-invasive approaches, such as total parenteral nutrition (TPN) (19, 24, 26).	2c
	3. The duration of TPN is recommended to range between 6 and 30 days (29).	4d
	4. For postoperative CL patients unable to consume food orally, nasogastric or gastrostomy tube placement with enteral feeding based on medium-chain fatty acid (MCT) should be initiated until CL resolves (29).	4d
4. Nutrient requirements	1. Oral multivitamins are recommended, with parenteral supplementation of these vitamins and trace elements considered if necessary (29, 30).	4c
	2. For patients at high risk of essential fatty acid deficiency, peripheral intravenous (IV) fat emulsion (250 mL of 20% emulsion/time, administered three times weekly) is recommended (30).	2c
5.Dietary adjustment strategies	1. For most CL patients with high leak volumes (>1 L/d) post-surgery, immediate discontinuation of oral feeding and initiation of TPN are recommended to ensure complete intestinal rest (24, 27, 29, 30).	2c
	2. For most CL patients with low leak volumes (< 1 L/d) after surgery, a low-fat and/or MCT diet is recommended. A low-fat diet should be maintained for 7–10 days (24, 27, 29, 30).	2c
	3. MCT can be administered orally in liquid or capsule form. It may be combined with juice for salads and vegetables, incorporated into sauces for fish, chicken, or lean meats, used in cooking or baking, or included in enteral feeding products (22, 30).	2c
	4. The recommended initial dose of oral MCT oil is 1 tablespoon, administered 3 to 4 times daily, with a typical adult dosage of 50–100 ml/day (31).	2c
	5. When administering MCT oil orally, adherence to a gradual progression approach is recommended, considering factors such as temperature, concentration, administration rate, cleanliness, and the angle of administration, while closely monitoring abdominal symptoms and patient complaints (22).	2c
	6. For patients unable to take MCT oil orally or with inadequate intake, or unable to reduce volume despite a standardized low-fat or MCT diet, TPN may be considered (19, 24, 28–30).	2c
	7. If the CL volume is < 50 ml, discontinuation of fasting treatment is recommended (24).	4a

## 4 Discussion

### 4.1 Nursing professionals should utilize scientific tools for nutritional risk screening, regularly monitor physiological functions in CL patients, and evaluate the feasibility and effectiveness of dietary interventions

Low PNI in cancer patients is associated with a higher incidence of postoperative CL (32), emphasizing the significance of nutritional status on patient outcomes. NRS 2002 and PG-SGA are recommended for nutritional assessment in cancer patients (21). Assessing nutritional status in CL patients involves evaluating changes in weight, triceps skinfold thickness, middle-arm circumference, and grip strength (23). Chylous fluid contains serum proteins, fat-soluble vitamins, minerals, and immune cells. During nutritional support, monitoring hepatorenal function, immunity, and electrolytes in CL patients is essential to prevent severe electrolyte imbalances and immune system impairment due to chylous fluid leakage (33). Early enteral feeding, an independent risk factor for CL (34), also promotes gastrointestinal recovery, shortens hospital stays, and does not elevate the risk of severe postoperative complications (35). Offered the present evidence, doctors and nursing professionals have to consider the advantages and dangers in specific scientific contexts to identify optimal postoperative feeding timing. A comprehensive evaluation of each patient's distinct scenarios, including their dietary requirements and the risks associated with various interventions, is important for maximizing postoperative treatment and enhancing outcomes. Dietary treatment needs analyzing cost, preference adjustment, and treatment outcome variability to enhance patient's compliance. This decision-making process is crucial, as inadequate or postponed nutrition provision can result in further damage of a patient's nutritional condition.

### 4.2 Optimizing dietary therapy timing and ensuring continuous nutritional support

Dietary therapy, as the most conservative treatment for CL, achieves full recovery in 66–100% of cases (36). Its effectiveness and practicality make it the preferred approach for cancer patients diagnosed with CL post-surgery (24). Postoperative malnutrition, driven by tumor-related consumption and hypermetabolism, increases the energy and protein needs of CL patients (37). Understanding the physiological mechanisms of chylous fluid production and the adverse effects of ongoing nutrient loss (38), continuous nutritional support is vital in CL management. Dietary fat restriction, which reduces chyle volume and aids healing, should be maintained for 2–4 weeks post-resolution of CL to prevent recurrence (26).

### 4.3 Establish MDT and determine appropriate nutritional treatment strategies based on feeding approach

The MDT model has become a prominent strategy in cancer diagnosis and treatment, providing significant benefits through integrated consultation during tumor care (39). Due to the complexities of CL management, forming MDTs is advised. Regular consultations with surgeons and nutritionists are essential to develop personalized dietary management plans tailored to the patient's condition, focusing on a high-protein, low-fat diet (29, 30). The definition of a low-fat diet remains controversial in clinical practice, with many patients finding it unpalatable. To improve palatability and reduce the risk of malnutrition, a low-fat diet can be supplemented with MCT, fasting, and TPN. Despite the absence of guidelines on optimal dietary combinations (38), healthcare professionals should evaluate and adjust the nutritional strategy daily based on the patient's condition and preferences. When TPN is utilized, its duration should be limited to 6–30 days to manage potential IV catheter-related complications and high costs (29). For postoperative CL patients unable to consume food orally, enteral feeding with MCT via a nasogastric or gastrostomy tube should be initiated and continued until CL resolves (40).

### 4.4 Assess nutritional status and supplement multiple nutrients as needed

In cancer patients, vitamins regulate calcium and phosphate metabolism, modulate cell proliferation and differentiation, reduce inflammation, and alleviate adverse reactions from cancer treatment. Additionally, they serve as independent indicators of frailty and mortality (37, 41). A fat-free or low-fat diet can hinder the absorption of various vitamins, and the lack of regular parenteral micronutrient supplementation may result in wound dehiscence in CL patients. Therefore, oral multivitamins are recommended, with parenteral supplementation administered as necessary (29). Fatty acids play a role in reducing postoperative infectious complications and shortening hospital stays while maintaining nutritional balance (42). In cases of high risk for essential fatty acid deficiency, a peripheral IV fat emulsion is recommended three times weekly at 250 mL of 20% solution per administration (30).

### 4.5 Regularly monitor CL volume, thoroughly assess patient symptoms, and promptly adjust dietary strategies

Total triglyceride output in chylous fluid accurately measures leak volume (43). Dietary treatment modifications should be based on chylous volume. Although CL volume classification is debated, many studies define a CL volume exceeding 1,000 mL within 24 h as high leak volume and < 1,000 mL as low leak volume (44). Post-surgical patients with high leak volume often enter a heightened metabolic state and may rapidly dehydrate, necessitating precise calculations for daily energy, protein, and fluid replenishment. TPN effectively reduces CL volume, promotes leak resolution, and prevents secondary surgery (45). Oral feeding can increase chylous volume, necessitating the prompt initiation of TPN to ensure complete bowel rest for patients with high leak volumes (30). For those with low postoperative CL volume, a high-protein, low-fat diet is recommended, emphasizing fats from MCTs. This diet should be maintained for 7–10 days (30). MCT is well-tolerated and can be administered orally in liquid or capsule form. MCT oil can be incorporated into various foods, such as mixed with juice for salads and vegetables, added to sauces for fish, chicken, or lean meats, or used in cooking and baking. Additionally, MCT can be included in enteral feeding products. The typical daily dosage for adult ranges from 50 to 100 mL starting with 1 tablespoon taken 3–4 times daily. Higher doses may cause steatorrhea, mild gastrointestinal discomfort, and elevated serum cholesterol in patients with hyperlipidemia (46). Administration should be gradually increased, considering factors such as temperature, concentration, rate, cleanliness, and angle, while closely monitoring the patient's abdominal symptoms and complaints (22). Continuous CL significantly elevates mortality rates in postoperative patients (47). For those unable to consume food orally post-surgery or with insufficient intake, and who do not achieve volume reduction despite a standardized low-fat or MCT diet, TPN may be considered (28). Given the potential for substantial fluid loss from leakage, daily monitoring of fluid balance is essential for CL patients. Fasting treatment should be discontinued if the CL volume falls below 50 mL (24).

### 4.6 Strengths and limitations

Our review presents innovative insights into managing cancer patients with CL. It proposes a structured approach for real-time nutritional adjustments tailored to individual patient needs and clinical feedback. These evidence enhances current understanding and offer practical strategies to improve patient outcomes and reduce postoperative complications. However, there are limitations to our review that must be acknowledged. The diversity in patient populations, types of cancer, and variability in clinical practices across different healthcare settings may influence the generalizability of our recommendations. Moreover, some innovative treatment strategies might not have been included in our evidence-based recommendations due to the current inability to fully establish their scientific validity, general applicability, and precise effectiveness. This limitation underscores the necessity for ongoing research and evaluation to substantiate these emerging approaches.

## 5 Conclusion

This study outlines the best evidence for nutritional management in cancer patients with CL post-surgery, focusing on five key areas: nutritional screening, assessment and monitoring, timing of nutritional therapy, methods and approaches of nutritional therapy, and nutrient requirements with dietary adjustment strategies. Our research highlights the crucial importance of nutritional management in improving the outcomes for cancer patients who experience CL. We highlighted the necessity of thorough nutritional screening and assessment through established tools such as the Nutritional Risk Screening 2002 (NRS 2002) and the Patient-Generated Subjective Global Assessment (PG-SGA). Ongoing assessment of physiological parameters and nutritional status, including changes in weight and muscle strength, is critical for alleviating the risks linked to CL. The present study emphasized the significant benefits of early enteral feeding as an intervention that not only supports gastrointestinal recovery but also reduces hospital stays without increasing the risk of serious complications. Furthermore, multidisciplinary team (MDT) approach supports the creation of personalized dietary management plans tailored to the unique needs and preferences of each patient. Moreover, we identified the importance of sustained nutritional support and dietary fat restrictions in the effective management of CL, particularly in preventing nutrient loss and promoting healing. The incorporation of medium-chain triglycerides is recommending as a notably effective strategy to enhance nutrient absorption while alleviating the adverse impacts associated with a low-fat diet. Finally, our research suggests the consistent monitoring of chylous fluid output, along with prompt adjustments to dietary strategies based on fluid volumes. This practice is crucial for enhancing patient management and promoting recovery. The practical implementation of these insights offers the potential for significantly improving clinical outcomes for patients dealing with CL, thereby underscoring the importance of personalized nutritional strategies within the realm of cancer care.

## Data Availability

The data analyzed in this study is subject to the following licenses/restrictions: this study is a secondary analysis of the existing literature, original results are available from the corresponding author. Requests to access these datasets should be directed to Xia Zheng, zhengxia@sysucc.org.cn.
