# Supplementary material for: Best evidence summary for nutritional management of cancer patients with chyle leaks following surgery
Source: Front Nutr. 2025 Jan 8;11:1478190. doi: 10.3389/fnut.2024.1478190 (PMC11752749; doi:10.3389/fnut.2024.1478190)
Supplement: Supplementary file 1 [file Table_1.DOCX]

**Supplementary Material. PubMed search strategy**

#1 "cancer"[Title/Abstract] OR "carcino*"[Title/Abstract] OR "tumo*"[Title/Abstract] OR "neoplasm*"[Title/Abstract] OR "onco*"[Title/Abstract]

#2 "Neoplasms"[MeSH Terms]

#3 #1 OR #2

#4 "Chyle"[Title/Abstract] OR "chyle leakage"[Title/Abstract] OR "chylous fistula"[Title/Abs-tract] OR "chyle fistula"[Title/Abstract] OR "chylous ascites"[Title/Abstract] OR "chyle lea-ks"[Title/Abstract] OR "Chylothorax"[Title/Abstract] OR "milk leakage"[Title/Abstract]

#5 "Chyle"[MeSH Terms] OR "Chylous Ascites"[MeSH Terms] OR "Chylothorax"[MeSH Terms]

#6 #4 OR #5

#7 "nutrition*"[Title/Abstract] OR "nutrition status"[Title/Abstract] OR "Nourishment"[Title/Abstract] OR "nutrition management"[Title/Abstract] OR "nutritional support"[Title/Abstract] OR "diet therapy"[Title/Abstract] OR "diet"[Title/Abstract] OR "enteral nutrition"[Title/Abst-ract] OR "enteral feeding"[Title/Abstract] OR "tube feeding"[Title/Abstract] OR "parenteral nutrition"[Title/Abstract] OR "nutrition disorders"[Title/Abstract]

#8 "Nutritional Support"[MeSH Terms] OR "Diet Therapy"[MeSH Terms] OR "Diet"[MeS-H Terms] OR "Enteral Nutrition"[MeSH Terms] OR "Parenteral Nutrition"[MeSH Terms]

#9 #7 OR #8

#10 "guideline"[Title/Abstract] OR "guideline*"[Title/Abstract] OR "consensus"[Title/Abstra-ct] OR "systematic review"[Title/Abstract] OR "Meta-analysis"[Title/Abstract] OR "evidenc-e summary"[Title/Abstract] OR "randomized controlled trial"[Title/Abstract]

#11 #3 AND #6 AND #9 AND #10
